# Early experience with the Venus p‑valve for percutaneous pulmonary valve implantation in native outflow tract

**DOI:** 10.1007/s12471-016-0932-5

**Published:** 2016-12-09

**Authors:** F. Garay, X. Pan, YJ. Zhang, C. Wang, D. Springmuller

**Affiliations:** 1 Departamento de Cardiología Pediátrica y Enfermedades Respiratorias, Hospital Clínico de la Universidad Católica de Chile, Pontificia Universidad Católica de Chile, Santiago Chile, Chile; 2 Cardiovascular Department, Shanghai Chest Hospital, Shanghai Jiao Tong University, Shanghai, China

**Keywords:** Pulmonary regurgitation, Percutaneous pulmonary valve implantation, Native right ventricle outflow tract, Venus p‑valve

## Abstract

**Introduction:**

The Venus p‑valve (MedTech, Shanghai, China) is a self-expanding percutaneous heart valve designed to be implanted in a native patched right ventricle outflow tract. The worldwide clinical experience with this valve is just beginning and the results have so far been encouraging. We present our initial early experience implanting the Venus p‑valve in the native right ventricle outflow tract of patients with Tetralogy of Fallot repaired with a transannular patch.

**Methods:**

In 10 selected patients a procedure for percutaneous pulmonary valve implantation was performed using the Venus p‑valve. The patients mean age was 32 years (13–57), mean weight 59.6 kg (40–80). All patients had Tetralogy of Fallot with moderate to severe pulmonary regurgitation and an indication for pulmonary valve replacement.

**Results:**

The implantation procedure was successful in all the patients resulting in an immediately functional valve. No procedure-related complications were observed. Follow-up after 12 months (4–21) resulted in an improvement in NYHA class. There was a reduction of the mean right ventricle diastolic volume from 139 ml/m^2^ (105–179) to 78 ml/m^2^ (65–100) and improvement in the regurgitation fraction from 42% (29–58) to 1% (0–5), as seen on routine cardiac magnetic resonance 6 months after the implantation. No stent fractures have been observed so far.

**Conclusion:**

Percutaneous pulmonary valve implantation with the Venus p‑valve resulted in a safe and effective procedure. The valve has predictable and sustained functional competence, resulting in clinical improvement in the patients.

## Introduction

Percutaneous pulmonary valve implantation has become a safe and effective option for patients needing pulmonary valve replacement after surgical repair [1–3]. The currently available percutaneous valves (Melody or Sapien) are recommended to be implanted only in right ventricle outflow tracts (RVOT) constituted by homografts or conduits. However, most of the patients with Tetralogy of Fallot undergo a repair using a transannular patch technique which determines a dilated and distensible RVOT usually exceeding the diameter of the available percutaneous valves [4]. For these reasons there is a need for a valve that can be implanted in the native RVOT and is large enough for the diameters usually found in patched RVOT.

The Venus p‑valve (Venus MedTech, Shanghai, China) is a self-expanding percutaneous valve designed to be implanted in a native patched RVOT. It has already been tested [5, 6] with satisfactory and encouraging results but is still not CE certified or FDA approved. The aim of this study was to report our initial experience on the feasibility and results of percutaneous implantation of the Venus P‑valve in the pulmonary position in patients with native RVOT.

## Materials and methods

### Patients

Patients with Tetralogy of Fallot repaired using a transannular patch technique were considered for percutaneous insertion of a Venus p‑valve. Patients were selected based mostly on cardiac magnetic resonance (MRI) information when fulfilling two of the following inclusion criteria: moderate to severe pulmonary regurgitation and pulmonary regurgitant fraction >25%, right ventricular end-diastolic volume index >150 ml/m^2^, right ventricular ejection fraction <45%, NYHA class II or III symptoms and the pulmonary valve annulus or conduit size of >18 mm and <30 mm. Exclusion criteria were patients with body weight <30 kg, occluded central veins, unfavourable RVOT anatomy as aneurysmal dilation, tortuosity of the main pulmonary artery (MPA) and a pyramid-shaped RVOT.

All the patients were evaluated with transthoracic echocardiography to assess: right ventricular size and function, tricuspid regurgitation, right ventricular systolic pressure, RVOT gradient and the degree of pulmonary regurgitation. In particular, a short axis view was used to measure the diameters of the pulmonary valve annulus, the MPA and pulmonary artery branches as well the MPA length, which is particularly useful for selecting the length of the valve to implant. MRI was also performed to define the size and the anatomy of the RVOT and pulmonary artery, left and right ventricular volumes and function and to calculate the pulmonary regurgitant fraction. With this information (particularly from echocardiographic measurements) the manufacturing company ensures that three or four possible valve sizes are available to be used in the patients on the day of the procedure. The study was approved by the local Institutional Review Board at each participating institution. Informed consent was obtained from the patients or parents for all the patients.

### Valve and delivery system

**Fig. 1:**
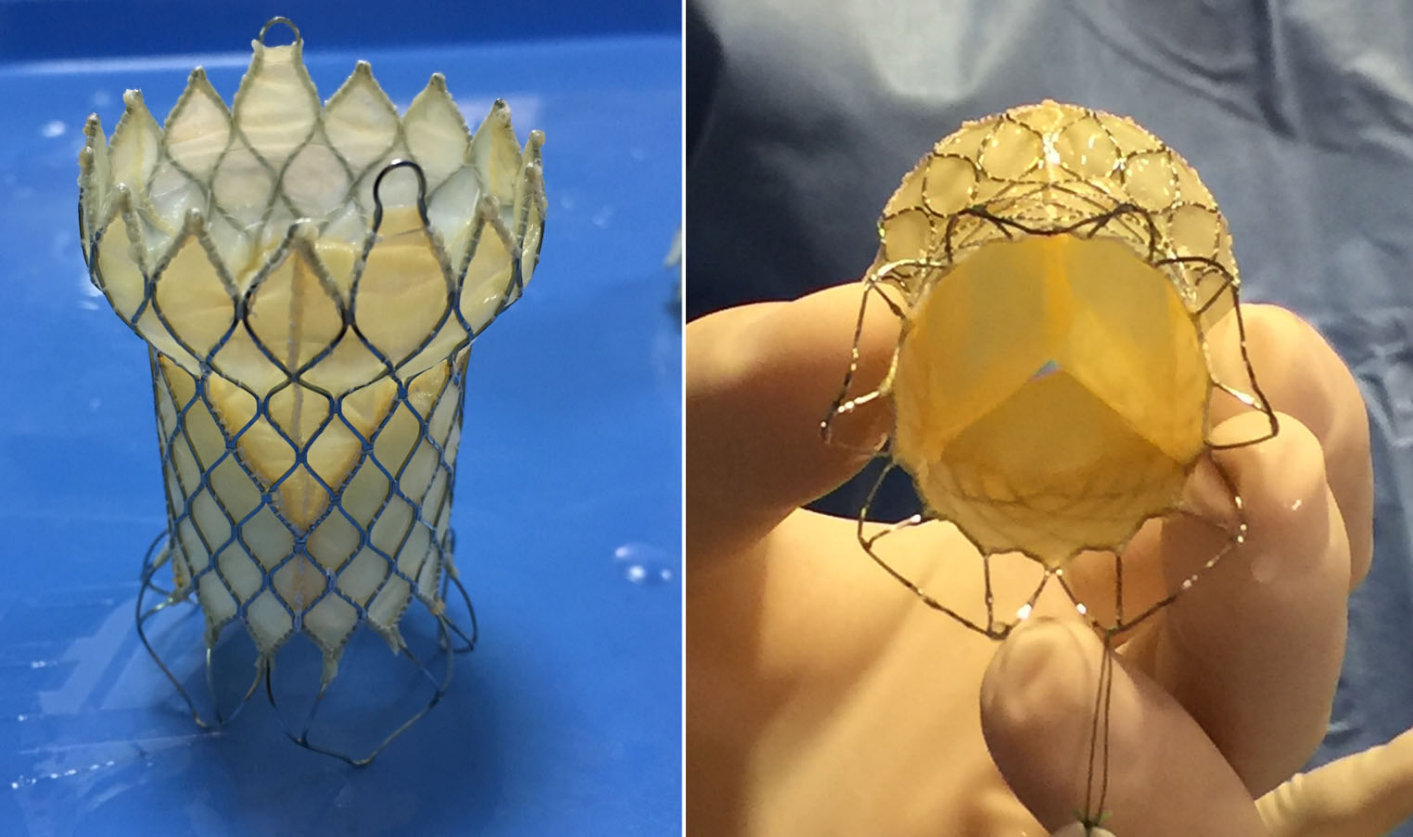
The Venus p‑valve. Note its particular design with flared ends, the non-covered distal end and the proximal hooks or ‘ears’ for attaching mechanism to the delivery catheter

**Fig. 2:**
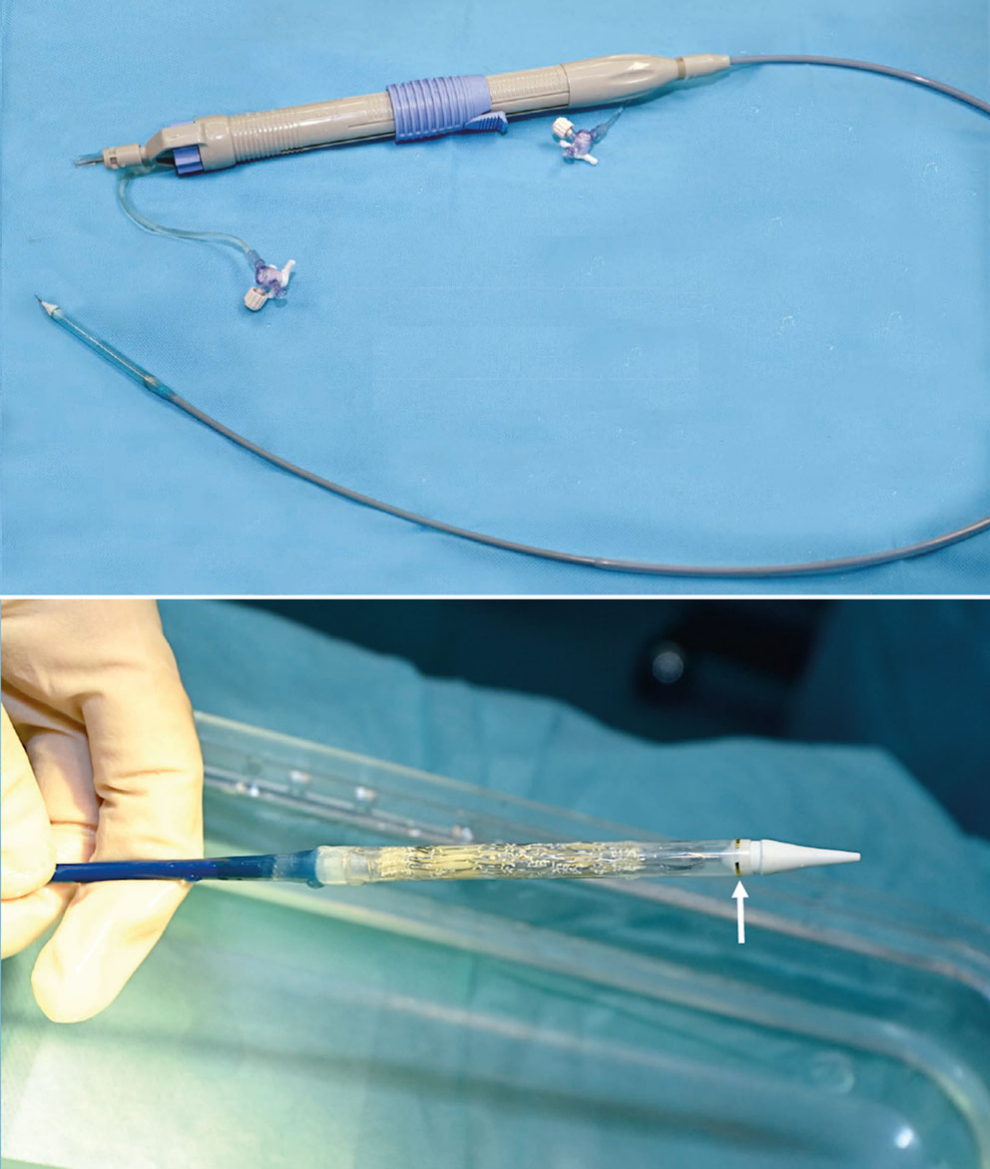
The Venus p‑valve delivery system. Note the handle with a knob for slow and controlled release of the valve and the distal capsule with a crimped and loaded valve inside. The *arrow* signs a distal radiopaque mark

The Venus P‑valve consists of a self-expanding stent made of nitinol with a tri-leaflet porcine pericardial tissue valve preserved in a low-concentration solution of buffered glutaraldehyde and hand-sewn inside of the nitinol frame (Fig. 1). The stent has proximal and distal flares to anchor the valve in the RVOT and in the pulmonary artery bifurcation. The proximal flare is covered by pericardial tissue, whereas the distal flare is an open cell wire frame to avoid obstruction of the pulmonary artery branches. The middle part is also fully covered by pericardium and the valve. There are three radiopaque platinum markers at the proximal flare to identify the valve location. The diameters of the middle part range from 18 to 34 mm with 2 mm increments and the lengths range from 20 to 35 mm with 5 mm increment. The proximal and distal flare diameters are 10 mm larger than the middle segment. There are two small hooks at the proximal part of the valve for attachment to the delivery system (Fig. 1). The delivery system consists of a 16 Fr 100-cm-long shaft catheter with a 20–22 Fr capsule and a handle rotating mechanism for controlled deployment of the valve (Fig. 2). The valved stent is crimped and loaded onto the delivery system under sterile cold saline solution which helps to reduce the memory property of nitinol. The delivery system is advanced through a 22–24 Fr sheath.

### Procedure

**Fig. 3:**
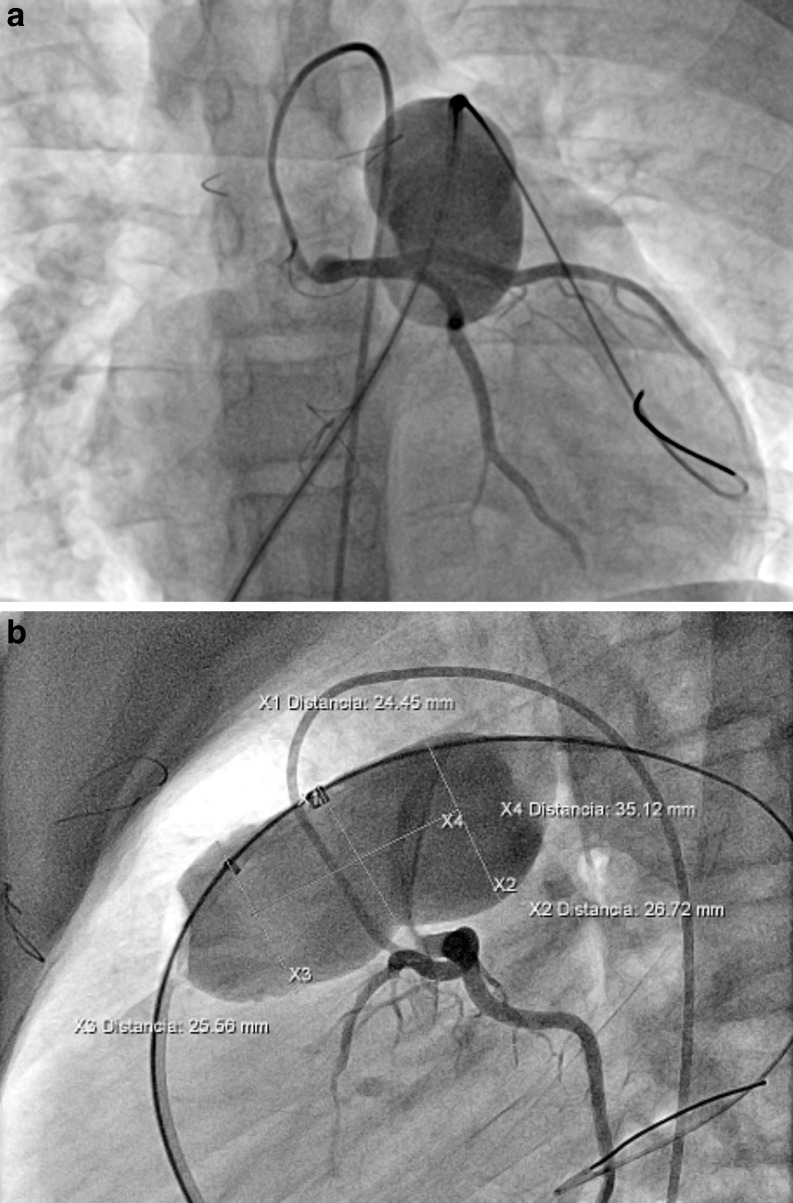
Sizing balloon interrogation of the right ventricle outflow tract in caudal projection (**a**) and lateral projection (**b**) with measurements included. Simultaneously selective left coronary angiogram is observed

All procedures were performed under general anaesthesia and biplane laboratory was used. Anticoagulation with heparin 100 UI/kg was routinely used at the beginning of the procedure. Intraprocedural cefazolin 100 mg/kg/day was given. Following a full basic haemodynamic assessment, angiograms were performed in the RVOT and MPA in order to characterise the shape and diameter of the RVOT and pulmonary artery bifurcation. Angiograms were usually performed in posteroanterior and lateral projections, and additionally in right anterior oblique with cranial angulation or left anterior oblique with cranial angulation for a better visualisation of the pulmonary artery bifurcation, since this understanding is key for the valve deployment. The coronary artery occlusion test was routinely performed with an aortic angiogram or selective left coronary angiography simultaneously with inflation of a 34 mm Amplatzer sizing balloon in the MPA to assess the expansibility and diameters as well as the proximity or compression of the left coronary artery (Fig. 3) which contraindicates the procedure.

**Fig. 4:**
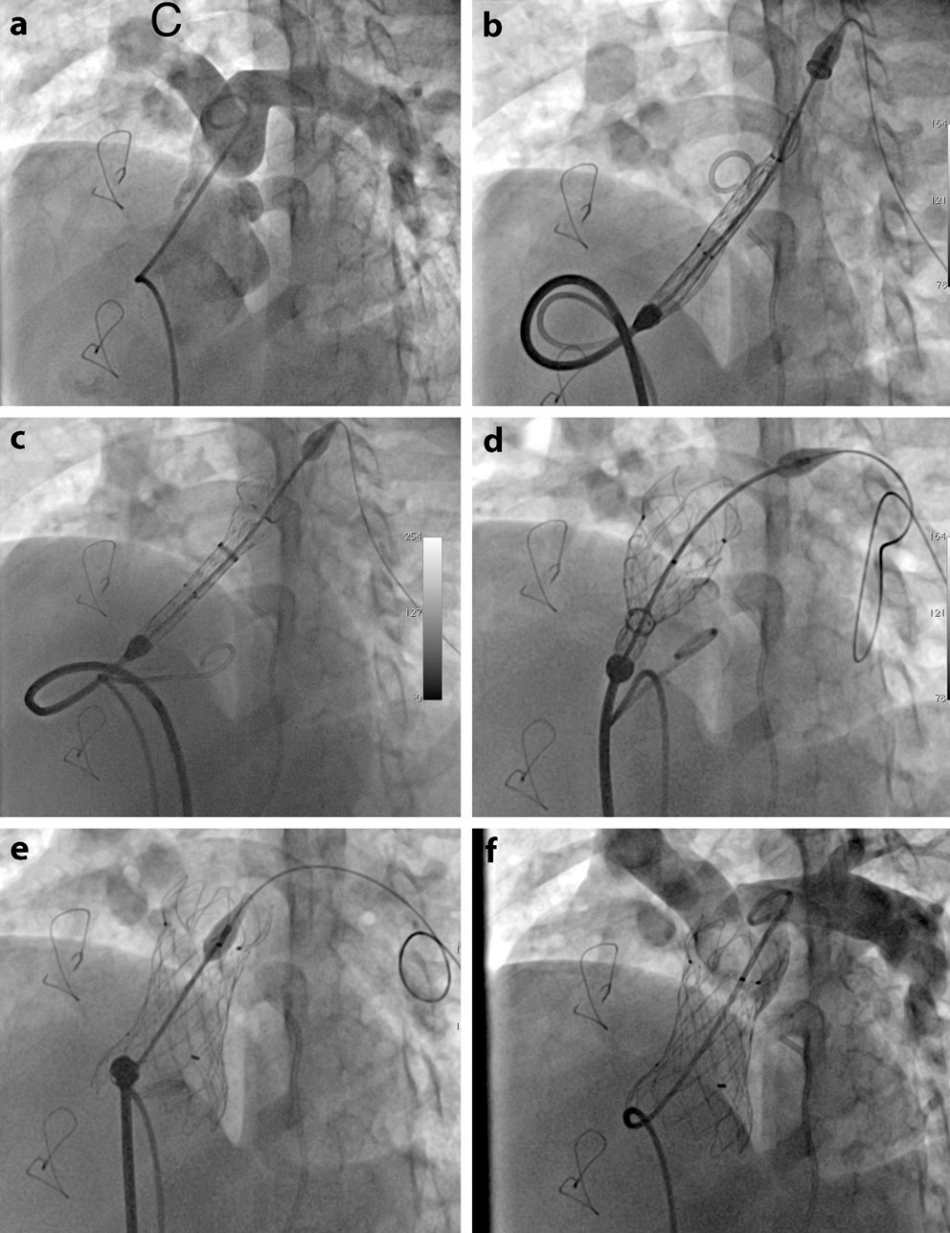
Fluoroscopic images demonstrating several steps of the implantation procedure. Basal angiogram in the main pulmonary artery in LAO and cranial projection to visualise pulmonary artery bifurcation (**a**), advancing and positioning Venus p‑valve delivery system in the RVOT with the tip advanced into proximal left branch (**b**), deployment of the distal end of the valve (**c**), deployment of the central part of the valve (**d**), Venus p‑valve completely deployed in an appropriate position (**e**) and final angiogram demonstrating a functional valve and patent pulmonary branches (**f**)

The selection of the valve size and length to implant was based on the minimum diameter of the pulmonary valve annulus/MPA during the sizing interrogation, selecting a valve diameter 3–4 mm larger than any waist in the sizing balloon. The valve length was selected to cover but not exceed the MPA length measured from the pulmonary valve annulus to the pulmonary artery bifurcation on transthoracic echocardiograph. Once the valve was prepared and loaded into the delivery catheter, it was advanced over an extra-stiff wire (260-cm, 0.035′ Lunderquist, Cook Medical) located distal in the left pulmonary artery. The valve was advanced into the main pulmonary artery observing the distal radiopaque markers of the valve being positioned just before the pulmonary artery bifurcation (Fig. 4). Then a slow and controlled valve deployment is performed by clockwise rotation of the knob in the handle. Repeated RVOT angiography is performed during the delivery to ensure an optimal position and that the distal end is not jailing any of the pulmonary artery branches. After valve deployment, evaluation is immediately performed including haemodynamic assessment, pulmonary artery angiography and transthoracic echocardiography. Haemostasis could be achieved by using a figure-of-eight suture, preclosure with the Abbott Perclose Proglide device or manual compression according operator preferences.

### Statistics

Continuous variables are expressed as means, whereas categorical variables are presented as numbers. Comparison of parameters before and after pulmonary valve implantation was analysed using Wilcoxon’s signed rank test. A *p*-value <0.05 was considered to be statistically significant.

## Results

Ten patients (7 female) were selected to receive a Venus p‑valve and it was successfully implanted in all of them. Their mean age was 32 years (range 13–57) and their mean weight was 59.6 kg (range 40–80) (Table 1). Functional class was NYHA II in 7 patients and NYHA III in 3 patients. All the patients had a basal cardiac MRI that showed a pulmonary valve annulus with a median of 22.7 mm (range 18 and 27 mm). The mean pulmonary regurgitant fraction was 42% (range 29–58%), mean right ventricular ejection fraction was 50.9% (range 33–81%) and mean right ventricular end-diastolic volume index was 139 ml/m^2^ (range 105–179 ml/m^2^). The mean MPA diameter measured by sizing balloon interrogation was 27 mm (range 21–30 mm). The mean length of the MPA was 28.1 mm ± 6.5 mm (range 21 to 38 mm). The implanted valve diameters ranged from 26 to 32 mm, whereas the selected implanted valve length was 25 mm in 7 and 30 mm in 3 patients. Immediate angiography in the MPA after valve implantation revealed a competent valve in all the patients. The mean diastolic pulmonary artery pressure increased from 11.4 to 18.9 mm Hg demonstrating a functional valve (Table 2). The pressure gradient across the RVOT decreased from 10.3 mm Hg (range 0–30) to 2.9 mm Hg (range 0–10) after the procedure. The mean fluoroscopy was 29 min (range 23.7–35). The radiation dose had a mean dose area product of 19,880 mGy*m^2^ (range 5317–23,454 mGy*m^2^) and a mean total air Kerma of 1598 mGy (range 218–2233 mGy). There were no procedure-related complications and no evidence of paravalvular leaks in any of the patients on transthoracic echocardiogram immediately after the procedure. The large profile of the delivery system did not result in any obvious vascular damage, nor did it prevent the advance of the valve through heart structures.

**Table 1:** Summary of the patients: demographics and preprocedural measurements

Patient	Age (years)	Weight (kg)	Height (cm)	Sex	Diagnosis	MPA diameter echo (mm)	MPA diameter MRI (mm)	MPA diameter sizing (mm)	MPA length echo (mm)	Diameter implanted Venus valve (mm)	Length implanted Venus valve (mm)
1	13	59	157	M	TOF	28	23	27	35	30	30
2	15	53	151	F	TOF	24	23	28	32	26	25
3	13	67	162	F	TOF	28	27	30	36	30	30
4	25	52	162	F	TOF	28	26	29	22	32	25
5	15	40	165	F	TOF	26	25	27	23	30	25
6	50	51	152	F	PVS	24	22	26	21	28	25
7	57	80	165	F	TOF	25	18	28	27	28	30
8	44	72	168	M	TOF	29	23	30	24	32	25
9	39	68	175	M	PVS	27	22	21	38	26	25
10	49	54	158	F	TOF	20	18	24	23	26	25
–	–	–	–	–	–	–	–	–	–	–	–
Mean	32	59.6	161.5	–	–	25.9	22.7	27	28	–	–

*TOF* Tetralogy of Fallot, *PVS* pulmonary valve stenosis, *MPA* main pulmonary artery, *RV* right ventricle, *PA* pulmonary artery

**Table 2:** Summary of the patients: haemodynamics and MRI information

	Basal RV-PA gradient (mm Hg)	Post RV-PA gradient (mm Hg)	Basal diastolic PA pressure (mm Hg)	Post diastolic PA pressure (mm Hg)	Basal RVDV (ml/m^2^)	Post RVDV (ml/m^2^)	Basal pulmonary regurgitation fraction (%)	Post pulmonary regurgitation fraction (%)
1	5	4	12	15	142	^a^	44	^a^
2	8	7	9	16	151	^a^	29	^a^
3	9	0	9	12	179	^a^	58	^a^
4	11	0	17	18	105	66	30	0
5	15	3	10	20	131	100	41	0
6	5	0	11	14	113	90	33	0
7	15	10	13	37	^a^	^a^	^a^	^a^
8	5	0	10	18	155	73	58	0
9	30	5	10	14	147	70	49	0
10	0	0	13	25	131	72	39	5
Mean	10.3	2.9	11.4	18.9	139	78*	42	1*

*RV* right ventricle, *PA* pulmonary artery, *RVDV* right ventricle diastolic volume

*p ≤ 0.05

^a^data not available

During a mean follow-up of 12 months (range 4–21) all the patients have remained in NYHA functional class I. Transthoracic echocardiography and MRI 6 months after implantation of the valve showed sustained and significant reduction of the pulmonary regurgitation in all the patients (Table 2). In 6 patients with MRI follow-up the median pulmonary regurgitant fraction was 1% (range 0–5%; significant at *p* ≤ 0.05) and the right ventricular end-diastolic volume index was 78 ml/m^2^ (range 66–100 ml/m^2^; significant at *p* ≤ 0.05). No stent fracture was demonstrated on fluoroscopic follow-up at 6 months.

## Discussion

The worldwide experience in implanting the Venus p‑valve is just beginning with as yet few published reports. We have reproduced the encouraging results reported by Cao and Prompham in 11 patients with Tetralogy of Fallot repaired using the transannular patch technique [5, 6]. We report an additional 10 patients with satisfactory results and have demonstrated a significant reduction of the right ventricular end-diastolic volume index and sustainable valve integrity on follow-up MRI. These results are similar to the early results with Melody and Sapien systems [1–3].

Before the currently available percutaneous pulmonary valves (Melody and Sapien) can be used in a native RVOT it is necessary to first implant a large stent to transform the native RVOT into a rigid conduit [7, 8]. For a native RVOT up to 26–27 mm, pre-stenting followed by implantation of any of these valves can be performed during the same procedure or in a staged approach. Also an RVOT reducer used with a self-expanding valve has been previously reported to address this problem, but it has not resulted in any development [9, 10]. The Venus p‑valve appears to be an option to address this scenario. This valve is currently available up to a maximum diameter of 34 mm, which is indicated for RVOT up to 30–32 mm. Prompham reported that 6 of 16 patients with the native RVOT and indication for pulmonary valve replacement could be selected to receive a Venus p‑valve [6]. Patients were excluded because of a severely dilated main pulmonary artery or unfavourable RVOT anatomy, such as short pyramid shaped RVOT. Selection of our patients based on MRI and echocardiographic measurements was effective since all of them underwent a successful procedure.

During the procedure, the deployment and positioning of the distal end of the valve must to be very precise so that it does not excessively protrude into the pulmonary artery bifurcation. To achieve this, radiopaque marks on the distal end of the straight part of the valve have to be positioned just at the pulmonary artery bifurcation. Repeated RVOT angiograms are used to help with this fine positioning. Some of these angiograms can be avoided when using 3DRA guidance and on screen 3D roadmap [11]. Haemodynamic monitoring is crucial during the deployment because this is a covered stent and when the valve is half deployed the blood flow through the RVOT can be compromised resulting in hypotension or bradycardia. The proximal end of the valve needs to be quickly deployed which results in a rapid recovery of haemodynamics and rhythm.

Incomplete detachment of the valve has been reported causing unintentional valve migration into the right ventricle [6]. In order to avoid this, attention must to be paid to the appearance of the proximal flare in two different fluoroscopic views checking the release from the proximal hooks. If one of these is not fully released gentle clockwise or counterclockwise rotation of the delivery system may allow the hooks to detach completely from the delivery system. Also, as a safety tip, the distal end (‘carrot’) of the delivery catheter must be pulled back carefully and under fluoroscopic imaging to rule out the theoretical risk of engaging the stent with this.

Stent fractures were not seen in our patients. This has been reported for the Melody valve and pre-stenting has reduced this [12]. For Venus p‑valve implantation, pre-stenting is not necessary but it is possible that a dilated non-calcified native RVOT exerts less compression force on the stent frame avoiding the occurrence of stent fractures. However, the proximal end of the Venus valve is deployed in the contractile RVOT, this being a factor to produce stent fractures. Follow-up studies will be needed to evaluate the occurrence of late fractures.

## Conclusions

Our experience reproduces the encouraging initial results of implanting the Venus p‑valve in patients with Tetralogy of Fallot repaired using the transannular patch technique. The Venus p‑valve is expanding the scope of percutaneous valve implantation in patients with a dilated RVOT which is out of range for the available percutaneous valves. The Venus p‑valve seems to work well in the short term follow-up. Further long-term studies are needed to bring more information on issues like durability and fractures.
